# Perceptions of anti-racism efforts and mental health among students in higher education in the United States

**DOI:** 10.3389/fpubh.2025.1529835

**Published:** 2025-02-07

**Authors:** Hans Oh, Nicole R. Karcher, Megan Besecker, Jordan E. DeVylder, Karen D. Lincoln

**Affiliations:** ^1^Suzanne Dworak Peck School of Social Work, University of Southern California, Los Angeles, CA, United States; ^2^Department of Psychiatry, School of Medicine, Washington University in St. Louis School of Medicine, St. Louis, MO, United States; ^3^Price School of Public Policy, University of Southern California, Los Angeles, CA, United States; ^4^Silver School of Social Work, New York University, New York, NY, United States; ^5^Department of Environmental and Occupational Health, Joe C. Wen School of Population and Public Health, University of California, Irvine, Irvine, CA, United States

**Keywords:** racism, anti-racism, depression, anxiety, psychosis, flourishing, college, suicide

## Abstract

**Background:**

Anti-racism efforts are imperative for campus communities, yet little is known about whether perceiving their presence on campuses relates to a range of mental health outcomes among students.

**Methods:**

We analyzed data from the Healthy Minds Study 2020–2021 (*N* = 110,203). Using multivariable logistic regression, we examined the associations between perceptions of anti-racism efforts and several mental health outcomes.

**Results:**

Individuals who disagreed that their schools combatted racism in their campus communities had significantly greater odds of mental health problems (depression, anxiety, psychotic experiences, suicidal ideation, suicide plan, suicide attempt, perceived need for help, and loneliness), and lower odds of flourishing when compared with those who strongly agreed with the statement. For most outcomes, we observed an apparent dose–response association.

**Conclusion:**

Perceiving the presence of anti-racism efforts on campuses was inversely associated with mental health problems, calling for more research to test the effects of anti-racism efforts on mental health.

## Introduction

Racism is a fundamental cause of health inequity that adversely impacts the mental health of people of color (1). With this in mind, the field of public health has increasingly been concerned with *anti-racism* (2, 3), which describes efforts to mitigate the effects of racism while working toward its elimination [see the work of (2, 4–7)]. Dei and Calliste define anti-racism as “an action oriented, educational and/or political strategy for systemic and political change that addresses issues of racism and interlocking systems of social oppression” (32, p. 188). Anti-racism efforts have aimed to promote knowledge, capabilities, and attitudes in higher education to enable faculty, administrators, staff, and students to actively dismantle racism in its many forms across campuses (8). Anti-racism represents a departure from multiculturalism and cultural competency that had previously prevailed in higher education discourse. While well-meaning, multiculturalism and cultural competency have often been critiqued for not emphasizing the power dynamics that play out across ethno-racial groups (9). In general, anti-racism efforts are still inchoate across institutions of higher education. However, some efforts have been coordinated through organizations such as the National Association of Diversity Officers in Higher Education (NADOHE), which aims to promote a broad set of anti-racist organizational strategies in higher education in the United States, including faculty development initiatives (e.g., hiring a more diverse workforce, offering trainings to reduce microaggressions in the workplace) and student development initiatives (e.g., recruiting under-represented students and offering scholarships). Little is known about which anti-racist organizational strategies can improve mental health outcomes in higher education settings among people of color.

Furthermore, the impact of anti-racist policies and interventions on mental health is largely unexamined. A convincing body of evidence shows that racial discrimination is associated with an increased risk for anxiety, depression, suicidal thoughts and behaviors, and overall health (10), and an emerging body of evidence also suggests even vicarious discrimination can have similar effects (11, 12). Subjective measures of the environment have been linked to mental health (13, 14), including perceived racial biases and group-level discrimination (believing one’s racial group is being discriminated against), which have been associated with greater psychological distress (13). By extension, the perception that one’s institutional environment engages in anti-racism may also have an impact on mental health, though this has not been extensively studied.

Perceptions of racism in the environment could be linked to increased mental health problems linked to increased mental health linked to increased mental health problems by contributing to a sense of social defeat, whereby social exclusion sensitizes contribute to a sense of social defeat, whereby social exclusion sensitizes the mesolimbic dopamine system to produce mental health problems such as psychotic experiences (15). In addition to a sense of social defeat, perceptions of racism may impart a sense of ‘thwarted belongingness’ where one does not feel connected to others, contributing to suicidal thoughts and behaviors (16). An emerging literature on vicarious racism illustrates the ways in which people do not need to directly experience racial discrimination to be impacted by it (12, 17). Witnessing racial discrimination happening to other people can produce vigilance and anticipatory stress (18).

It stands to reason that anti-racism may therefore be associated with reduced risk for mental health problems and that perceiving the presence of anti-racism programs alone can be beneficial. In this study, we explored the association between perceptions of anti-racism on campuses and a range of mental health outcomes among students in higher education. We hypothesized that perceiving the presence of anti-racism efforts would be negatively associated with the odds of reporting mental health outcomes.

## Methods

### Sample

The Healthy Minds Study is a non-probability online survey administered to students in higher education in the United States (19). The survey was administered at 140 institutions between September 2020 and June 2021. We limited the sample to emerging adults (aged 18–29) and used complete-case analysis. The final analytic sample was 110,203. The response rate for the survey was 14%. To account for non-response, we used sample probability weights [based on administrative data at each institution (19)]. HMS data were collected with the approval of Advarra and all participating colleges/universities (IRB number: Pro00028565). The secondary analysis presented in this study was deemed exempt under the approval of USC (UP-22-00068).

### Measures

#### Perceptions of anti-racism (predictor)

Perceptions of anti-racism (predictor) were measured using the single item: How much do you agree with the following statement? “I believe my school actively works towards combating racism within the campus community.” Response options included: *strongly agree, agree, somewhat agree, somewhat disagree, disagree, and strongly disagree.*

### Mental health (outcomes)

We examined several mental health outcomes. *Depression* was measured using the Patient Health Questionnaire-9 [PHQ-9; (20)], which captures the frequency of depression symptoms over the past 2 weeks. The items were summed into a scale (range: 0–27), which was dichotomized (cutoff: 10) to reflect moderately severe or severe depression (21). *Anxiety* was measured using the Generalized Anxiety Disorder-7 [GAD-7; (22)], which captures the frequency of anxiety symptoms over the past 2 weeks. The items were summed into a scale (range: 0–21), which we then dichotomized (cutoff: 10) to reflect moderately severe or severe anxiety in accordance with prior studies (23). Psychotic experiences were measured using an abbreviated version of the World Health Organization Composite International Diagnostic Interview Psychosis Screen, assessing the lifetime prevalence of subthreshold hallucinations and delusions (strange feeling, persecutory delusion, and thought control) (24). *Suicidal thoughts and behaviors* were measured by the dichotomous items (yes/no) eliciting past year suicidal ideation, suicide plan, and suicide attempt. *Perceived need for help* was measured using a single ordinal item about needing help for emotional/mental health problems (feeling sad, blue, anxious, or nervous), which was dichotomized to reflect general agreement or disagreement. *Loneliness* was measured using the 3-item UCLA loneliness scale (range: 3–9) (25). The scale was then dichotomized such that those who had scores of 6 or higher were deemed significantly lonely. Flourishing/Languishing was assessed using a scale (range: 8–56) that elicits the respondent’s level of agreement with eight statements about flourishing (26, 27). The flourishing scale was dichotomized such that a score of 47 or lower was considered languishing (28).

### Sociodemographic characteristics (covariates)

Respondents reported gender (man, woman, non-binary/transgender/self-described), age (continuous), and race/ethnicity (White, Black, Latinx/Hispanic, Asian American/Pacific Islander, Multiracial, and Other).

### Analysis

We used multivariable logistic regression models to examine associations between perceptions of anti-racism (categorical) and the mental health outcomes of interest (depression, anxiety, psychotic experiences, suicidal ideation, suicide plan, suicide attempt, perceived need for help, loneliness, and flourishing). We tested for dose–response associations by re-running all models while treating anti-racism as continuous. We also conducted exploratory sensitivity analyses to determine whether race/ethnicity moderated the associations between perceptions of anti-racism and mental health outcomes, using interaction terms. We presented findings as odds ratios (ORs) with 95% confidence intervals (95% CIs). We used Stata SE 15 to perform all analyses.

## Results

Descriptive statistics are presented in Table 1 and have been presented in other studies (19). In response to the statement, *I believe my school actively works toward combating racism within the campus community,* approximately 15.3% of respondents strongly agreed, 34.2% agreed, 30.9% somewhat agreed, 10.4% somewhat disagreed, 6% disagreed, and 3.2% strongly disagreed.

**Table 1:** Descriptive statistics.

	Perceptions of Anti-racism							*p*-value
	Strongly agree	Agree	Somewhat agree	Somewhat disagree	Disagree	Strongly disagree	Total	
	(*n* = 14,234)	(*n* = 36,177)	(*n* = 35,931)	(*n* = 12,655)	(*n* = 7,560)	(*n* = 3,646)	(*n* = 110,203)	
Mental health outcomes								
Major Depression								<0.001
No	8,711 (64.3%)	21,502 (62.2%)	19,177 (55.9%)	5,840 (48.4%)	3,149 (43.8%)	1,286 (37.1%)	59,665 (56.7%)	
Yes	4,837 (35.7%)	13,082 (37.8%)	15,138 (44.1%)	6,219 (51.6%)	4,047 (56.2%)	2,179 (62.9%)	45,502 (43.3%)	
Generalized Anxiety								<0.001
No	9,190 (68.1%)	23,108 (67.2%)	21,297 (62.4%)	6,703 (55.9%)	3,651 (51.0%)	1,485 (43.6%)	65,434 (62.6%)	
Yes	4,301 (31.9%)	11,273 (32.8%)	12,815 (37.6%)	5,298 (44.1%)	3,507 (49.0%)	1,922 (56.4%)	39,116 (37.4%)	
Lifetime Psychotic Experiences								<0.001
No	9,516 (70.8%)	24,631 (71.7%)	23,672 (69.6%)	8,001 (66.9%)	4,615 (64.7%)	1,969 (57.8%)	72,404 (69.4%)	
Yes	3,930 (29.2%)	9,703 (28.3%)	10,347 (30.4%)	3,960 (33.1%)	2,514 (35.3%)	1,436 (42.2%)	31,890 (30.6%)	
Suicidal Ideation								<0.001
No	12,104 (89.2%)	30,630 (88.7%)	29,570 (86.4%)	10,064 (83.7%)	5,863 (81.8%)	2,654 (77.4%)	90,885 (86.6%)	
Yes	1,462 (10.8%)	3,916 (11.3%)	4,659 (13.6%)	1,960 (16.3%)	1,301 (18.2%)	774 (22.6%)	14,072 (13.4%)	
Suicide Plan								<0.001
No	12,956 (95.6%)	32,949 (95.5%)	32,386 (94.7%)	11,215 (93.4%)	6,625 (92.6%)	3,126 (91.4%)	99,257 (94.7%)	
Yes	597 (4.4%)	1,562 (4.5%)	1,809 (5.3%)	795 (6.6%)	528 (7.4%)	295 (8.6%)	5,586 (5.3%)	
Suicide Attempt								<0.001
No	13,407 (99.0%)	34,119 (98.9%)	33,768 (98.8%)	11,834 (98.6%)	7,046 (98.5%)	3,350 (98.0%)	103,524 (98.8%)	
Yes	138 (1.0%)	375 (1.1%)	423 (1.2%)	174 (1.4%)	107 (1.5%)	69 (2.0%)	1,286 (1.2%)	
Perceived Need for Help								<0.001
No	5,579 (42.5%)	12,920 (38.3%)	10,377 (31.1%)	2,812 (23.9%)	1,549 (22.1%)	739 (22.3%)	33,976 (33.2%)	
Yes	7,556 (57.5%)	20,800 (61.7%)	23,025 (68.9%)	8,976 (76.1%)	5,464 (77.9%)	2,578 (77.7%)	68,399 (66.8%)	
Flourishing/Languishing								<0.001
No (Languishing)	6,570 (47.5%)	21,402 (60.8%)	24,565 (70.2%)	9,302 (75.8%)	5,630 (76.8%)	2,593 (73.6%)	70,062 (65.4%)	
Yes (Flourishing)	7,256 (52.5%)	13,796 (39.2%)	10,421 (29.8%)	2,965 (24.2%)	1,704 (23.2%)	931 (26.4%)	37,073 (34.6%)	
Loneliness								<0.001
No	6,835 (51.0%)	16,032 (46.8%)	13,536 (39.8%)	4,090 (34.2%)	2,321 (32.6%)	1,070 (31.4%)	43,884 (42.1%)	
Yes	6,574 (49.0%)	18,251 (53.2%)	20,452 (60.2%)	7,864 (65.8%)	4,805 (67.4%)	2,335 (68.6%)	60,281 (57.9%)	
Sociodemographic characteristics								
Age	21.22	21.22	21.29	21.28	21.37	21.52	21.23	
Race/ethnicity								<0.001
White	8,897 (62.5%)	23,444 (64.8%)	21,693 (60.4%)	7,111 (56.2%)	4,019 (53.2%)	1,653 (45.3%)	66,817 (60.6%)	
Asian	1,611 (11.3%)	4,192 (11.6%)	4,470 (12.4%)	1,633 (12.9%)	963 (12.7%)	385 (10.6%)	13,254 (12.0%)	
Black	1,050 (7.4%)	2,335 (6.5%)	3,156 (8.8%)	1,400 (11.1%)	1,016 (13.4%)	740 (20.3%)	9,697 (8.8%)	
Hispanic	1,081 (7.6%)	2,410 (6.7%)	2,401 (6.7%)	857 (6.8%)	518 (6.9%)	271 (7.4%)	7,538 (6.8%)	
Two or More	1,193 (8.4%)	3,094 (8.6%)	3,455 (9.6%)	1,386 (11.0%)	845 (11.2%)	460 (12.6%)	10,433 (9.5%)	
Middle Eastern/Arab	257 (1.8%)	472 (1.3%)	484 (1.3%)	173 (1.4%)	140 (1.9%)	76 (2.1%)	1,602 (1.5%)	
Pacific Islander	29 (0.2%)	43 (0.1%)	47 (0.1%)	11 (0.1%)	10 (0.1%)	4 (0.1%)	144 (0.1%)	
American Indian/Alaskan Native	33 (0.2%)	68 (0.2%)	75 (0.2%)	27 (0.2%)	10 (0.1%)	11 (0.3%)	224 (0.2%)	
Missing/unknown	83 (0.6%)	119 (0.3%)	150 (0.4%)	57 (0.5%)	39 (0.5%)	46 (1.3%)	494 (0.4%)	
Gender Identity								<0.001
Man	5,126 (36.0%)	10,984 (30.4%)	9,094 (25.3%)	2,640 (20.9%)	1,614 (21.3%)	951 (26.1%)	30,409 (27.6%)	
Woman	8,890 (62.5%)	24,440 (67.6%)	25,480 (70.9%)	9,349 (73.9%)	5,393 (71.3%)	2,365 (64.9%)	75,917 (68.9%)	
Non-binary	201 (1.4%)	720 (2.0%)	1,316 (3.7%)	643 (5.1%)	528 (7.0%)	310 (8.5%)	3,718 (3.4%)	
Missing/unknown	17 (0.1%)	33 (0.1%)	41 (0.1%)	23 (0.2%)	25 (0.3%)	20 (0.5%)	159 (0.1%)	

*p-values by Chi2 test for binary/categorical variables.*

**Figure 1:**
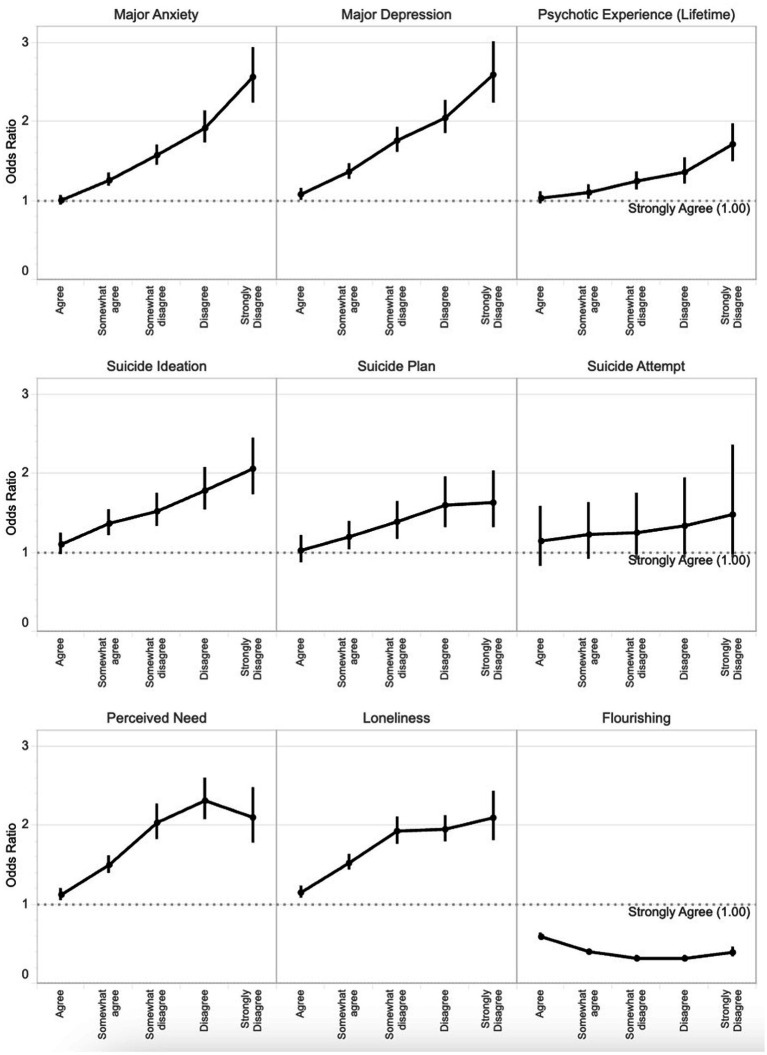
Dose–response associations between perceptions of anti-racism (reference: strongly agree to the statement “I believe my school actively works toward combating racism within the campus community”) and mental health outcomes. Vertical lines indicate 95% confidence intervals for the odds ratio.

Table 2 presents multivariable logistic regression models showing associations between perceptions of anti-racism and mental health outcomes. Individuals who reported disagreeing with this statement had significantly greater odds of mental health problems when compared with those who strongly agreed with the statement, adjusting for age, gender, and race/ethnicity. This was generally true for depression, anxiety, psychotic experiences, suicidal ideation, suicide plans, suicide attempts, perceived need for help, and loneliness. Those who strongly disagreed were over two and a half times as likely to report moderately severe to severe depression and anxiety. For flourishing, individuals who disagreed with this statement had significantly lower odds of flourishing (i.e., greater odds of languishing). For most outcomes, we observed an apparent dose–response association (Figure 1). Associations did not vary significantly with and without the inclusion of White students.

**Table 2:** Multivariable logistic regression models showing associations between perceptions of anti-racism and mental health outcomes.

		All races/ethnicities					White excluded				
Outcome	Response	Odds ratio	*p*-Value	LCI	UCI	Significant	Odds ratio	*p*-Value	LCI	UCI	Significant
Major Depression	Agree	1.08	0.021	1.01	1.16	Yes	1.14	0.022	1.02	1.27	Yes
	Somewhat agree	1.37	0.000	1.28	1.46	Yes	1.34	0.000	1.22	1.47	Yes
	Somewhat disagree	1.76	0.000	1.61	1.93	Yes	1.68	0.000	1.48	1.90	Yes
	Disagree	2.05	0.000	1.85	2.26	Yes	1.86	0.000	1.61	2.16	Yes
	Strongly Disagree	2.59	0.000	2.24	3.00	Yes	2.54	0.000	2.04	3.16	Yes
Generalized Anxiety	Agree	1.01	0.774	0.95	1.07	No	1.14	0.022	1.02	1.27	Yes
	Somewhat agree	1.26	0.000	1.18	1.35	Yes	1.34	0.000	1.22	1.47	Yes
	Somewhat disagree	1.58	0.000	1.46	1.71	Yes	1.68	0.000	1.48	1.90	Yes
	Disagree	1.92	0.000	1.73	2.13	Yes	1.86	0.000	1.61	2.16	Yes
	Strongly Disagree	2.57	0.000	2.24	2.94	Yes	2.54	0.000	2.04	3.16	Yes
Suicidal Ideation	Agree	1.11	0.092	0.98	1.24	No	1.14	0.204	0.93	1.39	No
	Somewhat agree	1.37	0.000	1.22	1.54	Yes	1.26	0.006	1.07	1.49	Yes
	Somewhat disagree	1.52	0.000	1.33	1.75	Yes	1.54	0.000	1.25	1.89	Yes
	Disagree	1.79	0.000	1.54	2.07	Yes	1.56	0.000	1.26	1.92	Yes
	Strongly Disagree	2.06	0.000	1.73	2.45	Yes	1.89	0.000	1.46	2.45	Yes
Suicide Plan	Agree	1.03	0.728	0.88	1.21	No	1.15	0.272	0.89	1.48	No
	Somewhat agree	1.20	0.014	1.04	1.39	Yes	1.20	0.090	0.97	1.48	No
	Somewhat disagree	1.39	0.000	1.17	1.65	Yes	1.50	0.002	1.16	1.94	Yes
	Disagree	1.60	0.000	1.31	1.95	Yes	1.37	0.013	1.07	1.76	Yes
	Strongly Disagree	1.63	0.000	1.32	2.03	Yes	1.55	0.011	1.11	2.17	Yes
Suicide Attempt	Agree	1.15	0.391	0.84	1.58	No	1.34	0.266	0.80	2.24	No
	Somewhat agree	1.23	0.153	0.93	1.64	No	1.09	0.689	0.72	1.63	No
	Somewhat disagree	1.25	0.180	0.90	1.75	No	1.20	0.440	0.75	1.93	No
	Disagree	1.34	0.116	0.93	1.93	No	1.36	0.156	0.89	2.08	No
	Strongly Disagree	1.48	0.093	0.94	2.35	No	1.57	0.147	0.85	2.90	No
Flourishing	Agree	0.59	0.000	0.56	0.63	Yes	0.57	0.000	0.51	0.64	Yes
	Somewhat agree	0.40	0.000	0.38	0.43	Yes	0.41	0.000	0.36	0.46	Yes
	Somewhat disagree	0.32	0.000	0.29	0.35	Yes	0.35	0.000	0.30	0.40	Yes
	Disagree	0.32	0.000	0.29	0.36	Yes	0.37	0.000	0.31	0.43	Yes
	Strongly Disagree	0.40	0.000	0.34	0.46	Yes	0.42	0.000	0.34	0.52	Yes
Loneliness	Agree	1.15	0.000	1.08	1.23	Yes	1.19	0.006	1.05	1.34	Yes
	Somewhat agree	1.52	0.000	1.43	1.62	Yes	1.48	0.000	1.34	1.63	Yes
	Somewhat disagree	1.93	0.000	1.76	2.11	Yes	1.90	0.000	1.64	2.21	Yes
	Disagree	1.95	0.000	1.79	2.13	Yes	1.65	0.000	1.40	1.93	Yes
	Strongly Disagree	2.10	0.000	1.81	2.43	Yes	1.99	0.000	1.63	2.44	Yes
Perceived Need for Help	Agree	1.13	0.001	1.05	1.21	Yes	1.28	0.001	1.12	1.47	Yes
	Somewhat agree	1.50	0.000	1.40	1.61	Yes	1.53	0.000	1.36	1.73	Yes
	Somewhat disagree	2.04	0.000	1.82	2.27	Yes	2.02	0.000	1.68	2.44	Yes
	Disagree	2.31	0.000	2.07	2.59	Yes	2.22	0.000	1.88	2.63	Yes
	Strongly Disagree	2.10	0.000	1.78	2.47	Yes	2.05	0.000	1.63	2.57	Yes
Psychotic Experience (Lifetime)	Agree	1.04	0.307	0.97	1.11	No	1.08	0.117	0.98	1.20	No
	Somewhat agree	1.11	0.014	1.02	1.20	Yes	1.04	0.527	0.92	1.17	No
	Somewhat disagree	1.25	0.000	1.14	1.37	Yes	1.35	0.000	1.17	1.56	Yes
	Disagree	1.36	0.000	1.21	1.54	Yes	1.29	0.007	1.08	1.56	Yes
	Strongly Disagree	1.72	0.000	1.49	1.98	Yes	1.59	0.000	1.32	1.91	Yes
Psychotic Experience (12 months)	Agree	1.02	0.753	0.92	1.13	No	1.11	0.264	0.92	1.33	No
	Somewhat agree	1.13	0.018	1.02	1.26	Yes	1.14	0.103	0.97	1.34	No
	Somewhat disagree	1.40	0.000	1.24	1.58	Yes	1.58	0.000	1.27	1.95	Yes
	Disagree	1.47	0.000	1.28	1.68	Yes	1.49	0.001	1.17	1.90	Yes
	Strongly Disagree	1.56	0.000	1.33	1.82	Yes	1.47	0.001	1.19	1.82	Yes
Psychotic Experiences: Hallucinations	Agree	1.02	0.811	0.87	1.19	No	1.13	0.327	0.88	1.46	No
	Somewhat agree	0.99	0.859	0.86	1.13	No	1.02	0.867	0.83	1.24	No
	Somewhat disagree	1.17	0.075	0.98	1.38	No	1.24	0.113	0.95	1.61	No
	Disagree	1.16	0.176	0.93	1.45	No	1.23	0.179	0.91	1.66	No
	Strongly Disagree	1.40	0.002	1.13	1.73	Yes	1.40	0.024	1.05	1.89	Yes
Psychotic Experiences: Strange Feeling	Agree	1.03	0.376	0.96	1.11	No	1.04	0.433	0.94	1.16	No
	Somewhat agree	1.10	0.023	1.01	1.20	Yes	0.98	0.795	0.86	1.13	No
	Somewhat disagree	1.26	0.000	1.15	1.38	Yes	1.31	0.000	1.14	1.52	Yes
	Disagree	1.31	0.000	1.16	1.48	Yes	1.19	0.075	0.98	1.44	No
	Strongly Disagree	1.66	0.000	1.43	1.91	Yes	1.51	0.000	1.24	1.82	Yes
Psychotic experiences: Persecutory delusion	Agree	0.87	0.022	0.77	0.98	Yes	0.89	0.206	0.74	1.07	No
	Somewhat agree	0.98	0.764	0.86	1.12	No	0.97	0.774	0.81	1.17	No
	Somewhat disagree	1.18	0.037	1.01	1.37	Yes	1.17	0.185	0.93	1.48	No
	Disagree	1.22	0.037	1.01	1.48	Yes	1.18	0.251	0.89	1.57	No
	Strongly Disagree	1.52	0.000	1.27	1.83	Yes	1.37	0.015	1.06	1.75	Yes
Psychotic experiences: Thought control	Agree	0.86	0.039	0.75	0.99	Yes	0.89	0.348	0.70	1.14	No
	Somewhat agree	0.80	0.003	0.70	0.92	Yes	0.75	0.008	0.60	0.92	Yes
	Somewhat disagree	0.89	0.198	0.75	1.06	No	0.91	0.479	0.70	1.18	No
	Disagree	1.01	0.920	0.83	1.22	No	0.96	0.742	0.73	1.25	No
	Strongly Disagree	1.30	0.038	1.02	1.67	Yes	1.04	0.798	0.79	1.36	No

All models are adjusted for age, gender, and race/ethnicity. Reference = Strongly agree.

## Discussion

### Main findings

Racism is a pervasive force that adversely impacts the mental health of people of color, making anti-racism efforts imperative. However, anti-racist efforts are often lacking across institutions. We found that among students in higher education, perceiving the absence of anti-racism efforts within the campus community was associated with greater odds of endorsing a wide range of mental health problems in a dose-dependent fashion. The more strongly students disagreed with the statement that their schools actively combatted racism, the more likely they were to endorse mental health problems such as depression, anxiety, psychotic experiences, suicidal thoughts and behaviors, perceived need for help, and loneliness. We found that perceiving the absence of anti-racism programs on campus was associated with lower odds of flourishing. We also found that perceiving the absence of anti-racism efforts was associated with poorer mental health across all racial/ethnic groups, including White students, even though White students are not directly affected by systemic racism. This association may be a positive externality where the benefits of anti-racism ‘spill over’ to White students. Moreover, existing within more diverse, inclusive, and equitable spaces may be beneficial for mental health regardless of whether one is directly affected by racism.

### Explanation of findings

Racism is a multi-faceted construct that impacts mental health through multiple pathways (29, 30). Racism in its many forms is pervasive on campuses. Since racism is deleterious to mental health, it stands to reason that actively combatting racism would result in the improvement of mental health. We cautiously interpret findings given the cross-sectional nature of the data and the absence of experimental conditions. There is some question as to whether perceptions of anti-racism are adequate proxies for the actual presence of anti-racism programs (or at least the visibility of anti-racism programs) on campuses. We acknowledge that perceptions do not always accurately reflect reality. Still, it is possible that colleges and universities that actively combat racism on campus are more diverse, equitable, and inclusive, such that students experience less discrimination, oppression, and exclusion, which are strongly linked to mental health.

### Limitations

To our knowledge, this study is among the first to examine perceptions of anti-racism efforts on campuses and their associations with mental health among emerging adults in higher education. However, there were several limitations of this study. First, in terms of measurement, we relied on a single self-report item to measure perceptions of anti-racism efforts on campus. It is not clear how students interpreted ‘anti-racism’, as it refers to a range of actions, including measures to change individual attitudes and behaviors, but also policies to improve racial equity. The single item did not allow students to specify what aspects of anti-racism they were able to observe. Future studies can use more extensive subjective and objective measures of anti-racism efforts (e.g., counting the number of anti-racist associations, anti-racist policies, and anti-racist trainings). The combination of these measures would be important in reducing bias. Second, in terms of sampling, the data were collected using a non-probability sampling strategy that yielded a relatively low response rate (14%). We used survey weights to attempt to account for non-response; however, future studies should use probability sampling strategies with incentives to increase participation and reduce sampling bias. Third, in terms of design, the cross-sectional nature of the study did not allow us to make any causal claims. While perceiving the absence of anti-racism efforts may reflect hostile and invalidating socioenvironmental conditions that lead to mental health problems, it is also possible that people with mental health problems are either more likely to perceive their environments negatively or less likely to be able to detect anti-racism on their campuses due to their symptoms (e.g., social isolation). More experimental designs are needed to test the effects of various anti-racism efforts on mental health across multiple settings. Finally, the data were collected in 2020 during the COVID-19 pandemic and during the Black Lives Matter (BLM) movement to protest anti-Black racism. The BLM movement raised awareness of racism and brought anti-racism to the foreground of many campuses. Moreover, hate crimes against Asian Americans surged across the country, impacting mental health on campuses (31). The HMS data collection coincided with these historical events, which may have resulted in a period effect. Future research can examine the moderating effects of the location of the school, the ethno-racial composition of the student body, the size of the student body, and the political orientation of the student. It is possible that what constitutes significant anti-racism programming in one department may seem inadequate in another department. For instance, students in the field of social work may be more attuned to issues of racism and may feel more discontent with the amount of anti-racist programming when compared with students in other professional degree programs.

### Implications and conclusion

Perceptions of anti-racism on campuses were inversely associated with mental health problems. It is worth noting that sometimes anti-racism efforts can potentially generate ‘backlash’ that stokes more racism (2). Anti-racist strategies can include programs to empower and educate, but can also include programs to build alliances for advocacy and social justice/activism (4). Future experimental studies should explore the effects of anti-racism on mental health toward reducing mental health and service disparities affecting students of color, and which specific strategies prove to be most effective.

## Data Availability

Publicly available datasets were analyzed in this study. This data can be found here: https://healthymindsnetwork.org/hms/.
